# PAU Smart Seeder: a novel way forward for rice residue management in North-west India

**DOI:** 10.1038/s41598-024-62337-z

**Published:** 2024-05-23

**Authors:** Manpreet-Singh, A. T. Chaleka, Rajesh Goyal, Naveen Gupta, Arshdeep Singh, Manpreet Singh, Sandeep Sharma, Anoop Kumar Dixit, Anurag Malik, Nadhir Al-Ansari, Mohamed A. Mattar

**Affiliations:** 1 Department of Farm Machinery and Power Engineering, Punjab Agricultural University, Ludhiana, India; 2 Department of Soil Science, Punjab Agricultural University, Ludhiana, India; 3 Department of Renewable Energy Engineering, Punjab Agricultural University, Ludhiana, India; 4 Punjab Agricultural University, Regional Research Station, Bathinda - 151001, Punjab India; 5 Department of Civil, Environmental and Natural Resources Engineering, Lulea University of Technology, 97187 Luleå, Sweden; 6 Department of Agricultural Engineering, College of Food and Agriculture Sciences, King Saud University, P.O. Box 2460, Riyadh 11451, Saudi Arabia

**Keywords:** Mulching, Incorporation, Speed index, In-situ straw management, Agroecology, Sustainability

## Abstract

In winter, the paddy residues become wet during morning and late evening due to dew, which restricts the operation of sowing machines (Happy Seeder and Super Seeder) into paddy residues, as wet residues do not slide on furrow openers/tines. A PAU Smart Seeder (PSS) was developed and evaluated for a four-wheel tractor that can sow wheat with optimum crop establishment in combined harvested rice fields. The PSS were evaluated for its performance under varying straw load, forward speed, and rotor speed in terms of fuel consumption, field capacity, seed emergence, and grain yield. The crop establishment and wheat yield of PSS was also compared with the existing straw management machines Happy Seeder (HS) and Super Seeder (SS) under heavy paddy residue conditions. The effect of the straw load was more pronounced on dependent variables than the effect of the speed index. PSS performance was best at a forward speed of 2.6 km h^−1^, rotor speed of 127.5 rpm, and a straw load of 6 t ha^−1^. Average fuel consumption using PSS was lower than SS but higher than HS. Wheat emergence was higher by 15.6 and 25.7% on the PSS plots compared to HS and SS, respectively. Average wheat grain yield in PSS plots was significantly higher by 12.7 and 18.9% than SS and HS, respectively in one experiment, while the grain yield was similar for both PSS and HS in other experiments. PSS has a novel mechanism to manage paddy straw and simultaneously sow wheat into a heavy straw load (> 8 t ha^−1^) mixture of anchored and loose straw. In conclusion, PSS showed promise for in-situ management of rice straw as it eliminates most of the operational problems encountered by the existing seeders (HS and SS).

## Introduction

The rice–wheat (RW) is a major production system in the Indo-Gangetic Plains of India covering nearly 10.5 million hectares including 4.1 million hectares in the north-western (NW) states of the country^1^. In the early 1970s, the introduction of high-yielding rice and wheat cultivars, high inputs of chemicals, availability of machinery and highly subsidized electricity for irrigation, and provision of assured minimum support price were responsible for achieving food security in the country. Since the mid-1980s, the practice of manual harvesting has been replaced by the advent of combine harvesters that leave a heavy load of residue scattered and anchored residues on the field. Unlike wheat straw which is collected from the fields and used as fodder, rice straw is rich in silica content which makes it unsuitable for animal feed^2^. Due to the lack of alternative economic use of rice straw and a short window for preparing the land for the next crop (wheat), it is subjected to burning in open fields^3,4^. Rice residue burning has become a serious problem causing phenomenal environmental pollution with serious consequences on both human and animal health^5–7^. It is estimated that about 23 million tonnes of rice residue are burnt annually in the two NW states (Punjab and Haryana) of India^1^. Burning also results in the loss of precious carbon as well as nutrients present in the rice straw^8^.

In-situ residue management provides the most convenient mode of recycling organic carbon and nutrients, thereby positively impacting soil health and crop performance. The two sustainable options for in-situ management of straw are surface retention (mulching) and incorporation. For managing rice straw, the Turbo Happy Seeder (or Happy Seeder, HS) machine was developed, refined, and validated over several years under diverse farming systems to establish its significance^9^. The HS represented a breakthrough for direct drilling of wheat in the combined harvested rice fields and retaining rice residue as surface mulch in a single operation in NW India. Another technological development was the attachment of the Super straw management system (Super SMS), a simple loose straw chopping and spreading mechanism, that can be attached to the rear of the combine harvester, enabling uniform spreading of residue across the harvesting width^1,9^. The Super SMS enhances the efficiency of the HS and improves the establishment of wheat^1,10^. Recently, Keil et al.^11^ have reported that HS has the potential to eradicate the practice of rice residue burning due to its ability to sow wheat directly into large amounts of anchored and loose residues. The HS leads to significant savings in wheat production costs and the benefits in terms of time and water savings, improving soil health, and the societal benefits of reducing air pollution through avoiding burning^12,13^. Despite these benefits of the HS, its uptake by the farmers is much slower than expected. Some of the drawbacks of HS technology are; (i) non-uniform seeding depth, particularly on fine-textured soils leading to poor crop establishment, (ii) lack of confidence in farmers due to slow germination and poor visibility of seed rows initially, (iii) low field capacity when the rice straw is wet due to untimely rains or due to overnight dew in early morning hours thereby reducing working hours and field capacity, and (iv) occasional choking of seed and fertilizer delivery pipes when the rice straw is wet^9^.

Crop residues can be incorporated partially or completely into the soil depending on the tillage system. Implements like stubble shavers, mouldboard plough, disc harrows, and cultivators are generally used for residue incorporation^14^. However, in-situ incorporation of cereal residues with a high C/N ratio leads to temporary immobilization of soil and requires 3–4 extra tillage operations and the use of a straw chopper and also requires one additional irrigation, thus making it a cost and energy-intensive option^1,15^. Given the limitations of HS technology listed above, a high energy input seeder (requiring > 60 hp 4-wheel drive tractor) known as Super Seeder was developed by agriculture machinery manufacturers in Punjab, India for seeding wheat into rice residue in a single operation. Super Seeder is a rotavator with the provision of drilling seed and fertilizer. The outlook of the field after the use of Super Seeder is very pleasing to farmers as they are accustomed to viewing the wheat establishment in a clean field (free from rice residue). In 2020, the purchase and use of Super Seeders by the farmers outnumbered HS in NW India. However, Super Seeder has also its drawbacks such as it requires high energy input and defeats the objective of conservation agriculture as it incorporates a major fraction of residue in the soil leaving the surface plain. Results from several on-farm experiments (n = 159) conducted in NW India showed that on average net returns from wheat for Super Seeder were about 15% lower compared to HS (H.S. Sidhu, Borlaug Institute for South Asia, personal communication). Furthermore, higher weed intensity and wheat lodging on a large acreage has been reported in the case of super Seeder compared to wheat sown with HS.

Strip tillage is a conservation tillage system that uses minimum tillage by disturbing only the portion of the soil that is to contain the seed row^16^. A seeder that combines the benefits of both straw incorporation and straw mulching may be a better technology for residue management as an alternative to HS or in-situ incorporation in the RW system. The strip tillage seeding system tills the land in narrow strips (7.5 cm wide) in front of the furrow opener only and places seed and fertilizer in rows at a right depth in a single operation leaving the inter-row with complete residue cover^17,18^. The findings^19^ involved the development of a strip-till drill for sowing wheat in rice fields. The average wheat yield was similar for conventional till and strip-till drills on manually harvesting rice fields and after removal of the straw. Over the last decade, innovations have been made to design and develop a wide range of 2-wheel tractor (2WT) driven no-till seeding implements (e.g. lightweight Versatile Multi-crop Planter, VMP) for smallholders which permit reliable seeding into minimally disturbed soil with moderate levels (< 4.5 t ha^−1^) of anchored residue^20,21^. Evaluation of the 2WT-driven VMP showed promising results for the establishment of a range of crops in rainfed cropping systems in Bangladesh^20–22^. The research findings^22^ reported that loose straw accumulated on the rotary shaft and furrow openers of VMP. At present, there is no strip tillage machine available that can handle and sow wheat into 6.0 t ha^−1^ or more straw load of rice residues. The objectives of the present study were to develop a PAU Smart Seeder (PSS) for sowing wheat into combined harvested paddy fields and compare it with existing residue managing seeders. This paper also provides information on the on-station and on-farm field evaluation of PSS in terms of energy use, field capacity, and wheat yield in comparison with the other commonly used direct seeding machines (HS and SS) being used for sowing wheat in combined harvested rice fields in NW India.

## Results

### Effect of straw load and speed index on the performance of PAU Smart Seeder

#### Fuel consumption

**Figure 1:**
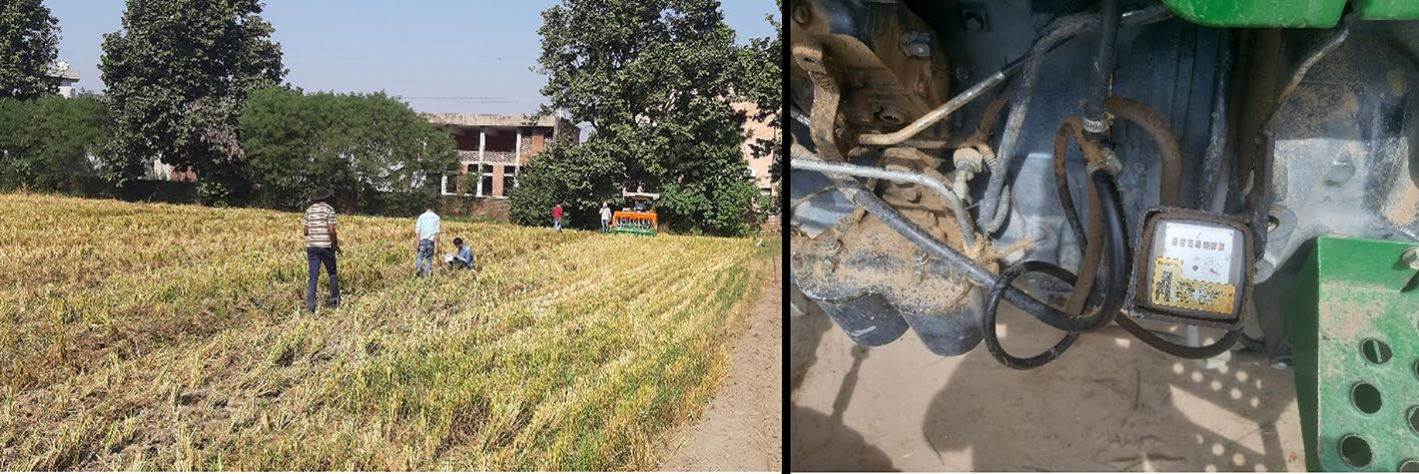
Test run of PSS to measure operational parameters like speed, slip and fuel consumption.

A significant increase in fuel consumption by the tractor (*John Deere 5310*, 50 hp) was observed in response to an increase in rice straw load during the field operation of PSS (Fig. 1, Table 1). Fuel consumption (FC_a_) by the tractor was 14.2 and 23.7% lower when PSS was operated under a straw load of 4.2 t ha^−1^ compared to a load of 5.2 t ha^−1^ and 6.0 t ha^−1^, respectively (Table 1).

**Table 1:** Effect of straw load and speed index on performance of Smart Seeder.

Treatment	Fuel consumption (FC_h_) (l h^−1^)	Fuel consumption (FC_a_) (l ha^−1^)	Effective field capacity (ha h^−1^)	Wheel slip (%)
A. Straw load (S)				
S1 (4.2 t ha^−1^)	4.41^a^* ± 0.24	12.19^a^ ± 0.62	0.36^a^ ± 0.02	− 0.94^a^ ± 0.27
S2 (5.2 t ha^−1^)	5.13^b^ ± 0.31	13.92^b^ ± 0.54	0.37^a^ ± 0.03	− 0.57^a^ ± 0.17
S3 (6.0 t ha^−1^)	5.73^c^ ± 0.29	15.08^c^ ± 0.66	0.38^a^ ± 0.03	− 0.68^a^ ± 0.20
LSD (0.05)	0.0003	< 0.0001	NS	NS
B. Speed Index (R)				
R1 (0.13)	4.69^a^ ± 0.17	15.89^c^ ± 0.50	0.30^c^ ± 0.01	− 0.43^a^ ± 0.27
R2 (0.20)	4.57^a^ ± 0.22	12.23^a^ ± 0.51	0.37^b^ ± 0.004	− 1.01^a^ ± 0.15
R3 (0.23)	6.00^b^ ± 0.33	13.08^b^ ± 0.48	0.46^a^ ± 0.01	− 0.76^a^ ± 0.19
LSD (0.05)	< 0.0001	< 0.0001	< 0.0001	NS
LSD (0.05) AxB	NS	NS	NS	NS

*Values followed by different letters in a column indicate significant differences between treatments at p ≤ 0.05. Values are mean ± standard error.

The increase in fuel (energy) consumption with increasing straw load was due to more work done by the strip-tillage rotor blades for incorporating the rice residue into the soil. Similarly, the speed index also significantly affected the fuel consumption by the tractor during the operation of the PSS. The lowest fuel consumption (4.57 l h^−1^ or 12.23 l ha^−1^) was observed under the speed index R2 (associated with the forward speed of 2.6 km h^−1^ and rotor speed of 127.5 rpm). An operating speed higher or lower than this optimum speed index of R2, resulted in significantly higher fuel consumption (l ha^−1^) (Table 1). This is consistent with the findings^23^ who recommended an optimum speed of 2.5 km h^−1^ for minimum fuel consumption, in the evaluation of a strip-till drill during wheat sowing. There was no significant interaction between straw load and speed index on fuel consumption during the operation of PSS.

#### Effective field capacity

The speed index significantly affected the effective field capacity of the PSS, whereas the amount of straw load had no significant effect on the field capacity (Table 1). Field capacity was lower (0.30 ha h^−1^) for the speed index R1 due to the lower forward speed of operation of the tractor (2.1 km h^−1^) as compared to the speed index of R2 and R3 where the tractor was operating relatively faster. The highest field capacity (0.46 ha h^−1^) was observed under speed index R3 (associated with the highest forward speed, 3.2 km h^−1^). Thus, field capacity increases when the speed of operation is increased and vice versa. The working video PSS sowing wheat into 10 t ha^−1^ paddy straw is available online at https://youtu.be/-8Y9TYneCY4.

#### Wheel slip

The wheel slip ranged from − 0.43 to – 1.01% but the values did not differ significantly with the increase in speed index (Table 1). The negative slip was due to the machine rotor rotating in the same direction as the direction of travel of the tractor, resulting in a pushing effect on the tractor. Therefore, the tractor covered more distance when the machine was in operation than the distance it would cover without the machine. The interaction effect of straw load and speed index on wheel slip during the operation of PSS was also not significant.

#### Wheat germination

Both straw load and speed index had a significant effect on seedling emergence of wheat sown using PSS (Table 2). The significantly lower emergence count was observed under the lower straw load of 4.2 t ha^−1^ compared to loads of 5.2 t ha^−1^ and 6.0 t ha^−1^ whereas the difference between the latter was not significant. The lower emergence under the lowest straw load may be due to the lower soil moisture content in the seeding zone at the sowing of the wheat. Residue mulch is known to reduce evaporation loss^4^ thereby enhancing soil water storage and facilitating early seed emergence. Straw load and speed index independently affected the emergence count; however, there was no significant interaction effect of straw load and speed index on the emergence count.

**Table 2:** Effect of straw load and speed index on plant germination, weed emergence and yield for PSS-sown wheat.

Treatment	Emergence count (m^−2^)	Weed density (weed count m^−2^)	Weed biomass (g m^−2^)	Grain yield (t ha^−1^)
A. Straw load (S)				
S1 (4.2 t ha^−1^)	131.2^b^* ± 3.4	27.7^c^ ± 1.1	29.7^c^ ± 1.2	3.77^c^ ± 174
S2 (5.2 t ha^−1^)	155.0^a^ ± 5.3	22.3^b^ ± 1.1	23.9^b^ ± 1.2	4.57^b^ ± 148
S3 (6.0 t ha^−1^)	157.4^a^ ± 3.6	18.7^a^ ± 1.0	17.9^a^ ± 1.1	5.03^a^ ± 93
LSD (0.05)	< 0.0001	< 0.0001	< 0.0001	< 0.0001
B. Speed Index (R)				
R1 (0.13)	159.0^a^ ± 5.8	21.4^a^ ± 1.7	23.0^a^ ± 1.9	4.28^a^ ± 220
R2 (0.20)	149.7^b^ ± 4.5	21.7^a^ ± 1.6	22.5^a^ ± 1.7	4.67^a^ ± 246
R3 (0.23)	145.0^b^ ± 5.2	20.6^a^ ± 2.0	22.0^a^ ± 2.2	4.42^a^ ± 211
LSD (0.05)	0.0029	NS	NS	NS
LSD (0.05) AxB	NS	NS	NS	NS

*Values followed by different letters in a column indicate significant differences between treatment means at p ≤ 0.05. Values are mean ± standard errors. *NS* non significant.

#### Weed density and biomass

Straw load significantly affected weed density and weed biomass, whereas speed index did not affect weed density and biomass (Table 2). Weed density was 19.6 and 48.2% lower in S3 (6 t ha^−1^) compared with S2 and S1 treatments (Table 2). The lower weed density under a high straw load may be due to the greater inhibition of weed seed germination and growth by causing the exclusion of sunlight from penetrating the high residue layer compared to a low residue load^4,24^. As a result, weed biomass was also lowest (17.9 g m^−2^) under S3 treatment and it was maximum (29.7 g m^−2^) when the straw load was lowest (S1).

#### Grain yield

The grain yield of wheat under high straw load (S3) was 33 and 10.3% higher compared to that under low straw loads S1 and S2, respectively (Table 2). It may be due to the higher seed germination and effective tillers under high straw load conditions. Speed index had no significant effect on the grain yield of wheat.

### Performance of the Smart Seeder in comparison with existing direct seeding machines during 2019–20 (Location:1)

#### Fuel consumption

**Figure 2:**
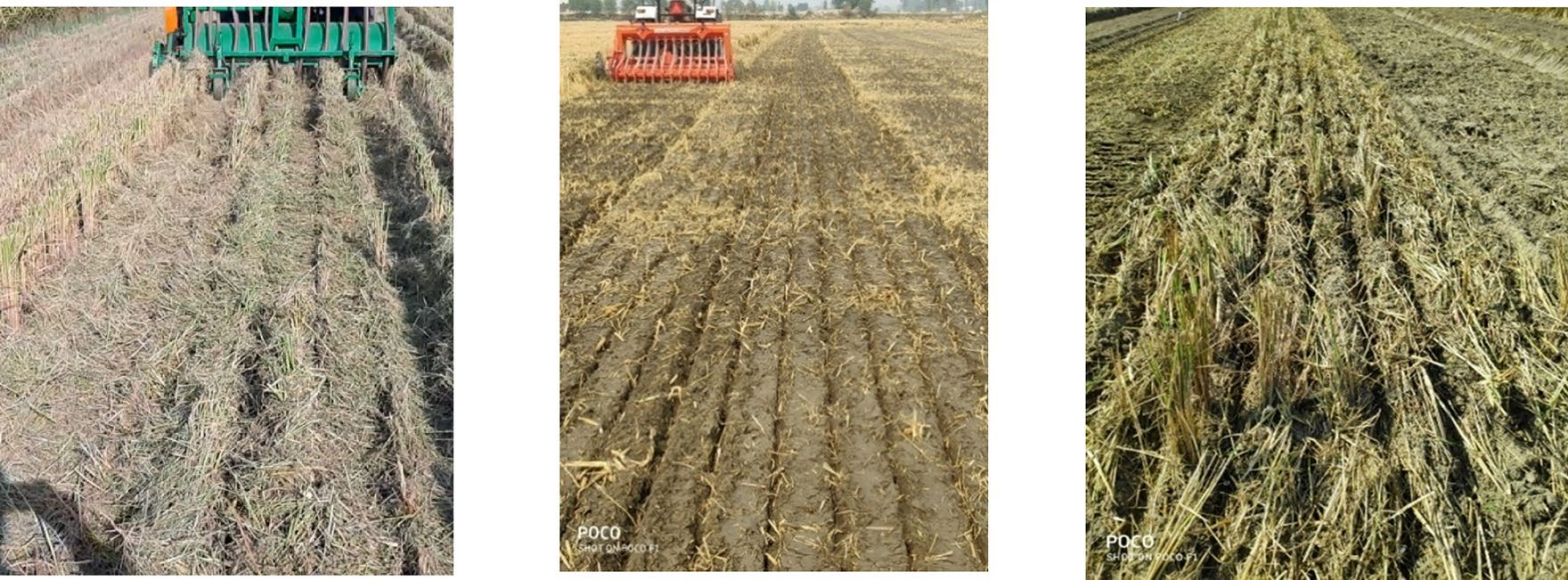
Field view after sowing with Happy Seeder (left), Super Seeder (Centre) and PAU Smart Seeder (Right).

Fuel consumption (l h^−1^) was significantly lower for HS than PSS and SS (Table 3). The average fuel consumption for PSS was 5.72 l ha^−1^, which was 23.3% lower compared to SS but it was 26% higher than HS (Table 3). These differences in fuel consumption were due to the differences in straw management mechanisms of the machines (Fig. 2).

**Table 3:** Comparison of operational parameters for different direct seeding machines.

Machine	Fuel consumption (l h^−1^)	Fuel consumption (l ha^−1^)	Effective field capacity (ha h^−1^)
PSS	5.72^b^* ± 0.29	14.98^b^ ± 0.65	0.38^a^ ± 0.025
SS	6.77^c^ ± 0.08	19.54^c^ ± 0.15	0.25^b^ ± 0.002
HS	4.50^a^ ± 0.11	11.88^a^ ± 0.10	0.38^a^ ± 0.008

*Values in a column with different letters are significantly different at p ≤ 0.05. Values are mean ± standard errors.

*PSS* PAU Smart Seeder, *SS* Super Seeder, *HS* Happy Seeder.

For example, while SS completely tills the soil while incorporating the entire residue into the soil, PSS partially tills the soil in front of the disc furrow openers only; the area between adjacent furrows is left undisturbed. The HS drills a narrow furrow (20 mm wide) during sowing compared to 75 mm for PSS. Thus, PSS combines both processes of incorporation and surface retention in one machine; therefore, the fuel consumption of PSS was in between HS and SS. However, effective field capacity (ha h^−1^) for all three machines was statistically similar (Table 3). In the case of PSS, the tilled 75 mm strip provides a pleasing look to the farmers compared to the field sown using HS. The roughness of the field after sowing using HS was a major bottleneck among the farmers for adoption of HS, despite having numerous advantages of surface mulch (Fig. 2). Moreover, the disc furrow openers of the PSS work fine in wet field conditions, whereas HS and SS machines face the problems of residue choking in the tine furrow openers.

#### Field capacity

The effective field capacity of PSS was 52% higher than SS but it was similar to HS (Table 3). The lower effective field capacity of SS was due to complete tillage and incorporation of rice residue into the soil. This is consistent with the earlier report^1^ suggesting that the incorporation of rice residue into the soil is an energy-intensive process.

#### Wheat emergence

The emergence of wheat seedlings was significantly higher in plots sown with PSS than that in HS and SS plots (Table 4, Fig. 1s). The number of seedlings at 20 days after seeding in PSS treatment was 15.5 and 25.6% higher compared to HS and SS, respectively (Table 4). The higher number of seedling emergence observed for PSS was mainly due to the fine seed zone soil tilth produced by the action of rotary blades that improved seed-soil contact facilitated by the furrow closing rollers. The emergence count in HS treatment was significantly higher than in SS. The lower seed emergence observed under SS may be due to the imperfect sowing depth mechanism of the machine. The SS incorporates 80–90% of rice straw into the soil and places the seed at the same depth of 5 to 7.5 cm. Therefore, seed germination and seedling emergence were low and less uniform in the case of SS. The fast rate of seed emergence and optimum plant stand are important parameters for the adoption of a residue management machine in the RW system of NW India. For this reason, the chances of adoption of PSS in the region are likely to be more compared with HS and SS.

**Table 4:** Comparison of plant germination, panicle length and weed emergence for different direct seeding machines.

Machine	Seedling emergence (plants m^−2^)	Weed density (weed count m^−2^)	Weed biomass (g m^−2^)	Grain yield (t ha^−1^)
PSS	157.5^a^* ± 3.6	16.7^a^ ± 1.0	17.9^a^ ± 1.09	5.03^a^ ± 0.093
SS	125.3^c^ ± 2.9	36.0^c^ ± 1.5	38.5^c^ ± 1.41	4.39^b^ ± 0.107
HS	136.3^b^ ± 5.9	26.3^b^ ± 1.1	28.4^c^ ± 1.30	4.08^b^ ± 0.031

*PSS* PAU Smart Seeder, *SS* Super Seeder, *HS* Happy Seeder.

Values in a column with different letters differ significantly at p ≤ 0.05. Values are mean ± standard errors.

#### Weed emergence and weed biomass

Weed density was significantly lower under PSS as compared to HS and SS. The average weed density at 30 days after seeding on PSS plots was 1.58 and 2.16 times lower than in HS and SS, respectively (Table 4). Similarly, weed biomass was significantly higher in SS and HS as compared to PSS. The lower weed density in PSS plots may be attributed to the combined effect of partial straw incorporation and the major portion of straw retained on the surface having a greater weed suppression effect compared to the effect of complete incorporation in SS, which exposed the weed seeds buried in lower soil layers. The early crop establishment in the case of PSS might have contributed to the greater suppression of weed growth compared to HS.

#### Grain yield

Grain yield was significantly higher by 14.6 and 23% on PSS plots compared to SS and HS, respectively (Table 4). However, there was no significant difference in grain yield between HS and SS treatments. Higher grain yield in PSS could be due to better crop establishment as indicated by higher emergence count and spike density compared with SS and HS. This is consistent with the findings^18^ who reported significantly higher wheat yield in strip-till sown wheat compared to conventional and minimum tillage systems.

### Evaluation of the PAU Smart Seeder in comparison with existing direct seeding machines during 2019–2020 (Location 2)

#### Wheat yield attributes and yield

The yield attributes of wheat for three seeders recorded at harvest showed that the average spike density (number per m row length) for PSS was significantly higher by 18.3 and 16.9% compared with SS and HS, respectively (Table 5). The higher spike density with PSS may be due to an increase in emergence count compared to sowing using HS and SS. The grain weight was also significantly more for PSS compared with HS and SS (Table 5). But both spike length and number of grains per spike were not influenced by the three sowing machines (Table 5). Wheat grain yield showed an increasing trend for PSS compared with SS and HS, however, the differences were not significant (Table 5).

**Table 5:** Effect of different sowing machines on yield attributing parameters (USF, Ladhowal).

Machines/treatment	Spike density (number m^−1^ row length)	Spike length (cm)	Grains per spike	1000-grain weight (g)	Grain yield (t ha^−1^)
PSS	97^a^ ± 1.1	10.9 ± 0.3	46.0 ± 1.9	40.0 ^a^ ± 0.3	4.84 ± 0.22
SS	82^b^ ± 2.1	11.6 ± 0.4	48.0 ± 5.5	39.0^b^ ± 0.0	4.68 ± 0.07
HS	83 ^b^ ± 2.5	10.6 ± 0.2	57.0 ± 6.1	39.2^b^ ± 0.2	4.43 ± 0.29
LSD	7.39	NS	NS	0.65	NS

± Standard error.

*PSS* PAU Smart Seeder, *SS* Super Seeder, *HS* Happy Seeder.

#### Effect on energy use

Total energy saving over farmer practice (conventional tillage system) was 66.8, 72.3, and 43.7% for using PSS, HS, SS, respectively (Table 6).

**Table 6:** Energy consumption in various operations for wheat establishment using PSS, HS and SS vis-à-vis farmers’ practice (FP).

Farm operation	Direct energy		Indirect energy		Total energy (MJ ha^−1^)	Energy saving over FP (%)
	Man (MJ ha^−1^)	Diesel (MJ ha^−1^)	Electricity (MJ ha^−1^)	Machinery (MJ ha^−1^)		
PAU Smart Seeder, PSS						
Super SMS attachment	2.0	140.8	0.0	2.7	145.5	
Land preparation	0.0	0.0	0.0	0.0	0.0	
Sowing	10.3	847.6	0.0	48.1	906.0	
Pre-sowing irrigation	0.0	0.0	0.0	0.0	0.0	
Tractor	0.0	0.0	0.0	34.1	34.1	
Rodent control	2.0	0.0	0.0		2.0	
Total	14.2	988.4	0.0	85.0	1087.6	66.8
Happy Seeder, HS						
Super SMS attachment	2.0	140.8	0.0	2.7	145.5	
Land preparation	0.0	0.0	0.0	0.0	0.0	
Tractor	0.0	0.0	0.0	34.1	34.1	
Sowing	10.3	666.8	0.0	46.8	723.9	
Pre-sowing irrigation	0.0	0.0	0.0	0.0	0.0	
Rodent control	2.0	0.0	0.0	0.0	2.0	
Total	14.2	807.6	0.0	83.6	905.4	72.3
Super Seeder, SS						
Super SMS	2.0	140.8	0.0	2.7	145.5	
Land preparation	0.0	0.0	0.0	0.0	0.0	
Sowing	15.7	1524.9	0.0	102.4	1643.0	
Tractor	0.0	0.0	0.0	51.8	51.8	
Pre-sowing irrigation	0.0	0.0	0.0	0.0	0.0	
Rodent control	2.0	0.0	0.0	0.0	2.0	
Total	19.6	1665.6	0.0	157.0	1842.2	43.7
Farmers’ practice (FP)						
Stubble shaver	2.5	315.3	0.0	3.9	321.7	
Residue burning	2.0	0.0	0.0	0.0	2.0	
Disc harrow	3.9	563.1	0.0	14.6	581.7	
Cultivator	3.9	563.1	0.0	6.3	573.3	
Planker	1.4	140.8	0.0	1.7	143.8	
Pre-sowing irrigation	19.6	0.0	1342.1	6.5	1368.2	
Sowing	5.0	252.7	0.0	9.2	266.9	
Tractor	0.0	0.0	0.0	16.6	16.6	
Total	38.3	1835.0	1342.1	58.8	3274.2	–

The high energy use for conventional till was due to pre-sowing irrigation and the associated energy consumption for pumping the groundwater (1368.2 MJ ha^−1^) and higher fuel consumption (1835.0 MJ ha^−1^) for residue management and tillage operations. The energy consumption for PSS and HS was 41.0 and 50.9% lesser compared to SS, respectively. While SS is a complete till system, PSS is a partial till (only seed row) machine and HS is a no-till seeder. Therefore, energy consumption for sowing wheat using PSS was in between HS and SS.

#### Evaluation of different seeders for sowing wheat into rice residue in 2020–2021

The wheat yield was 4.78, 4.73, 4.36, and 4.28 t ha^−1^ for HS, PSS, SS, and CT, respectively (Table 7). The average wheat yield of PSS over SS and CT was more by 8.5 and 10.6%, respectively. Grain yield was similar for both PSS and HS. The results from this study showed that the wheat yield of PSS was either more or at par with existing wheat sowing machines like HS and SS. The PSS will outperform the HS for sowing wheat in wet/heavy soils as heavy soil tends to choke the tines of HS. The furrow openers of PSS consist of disc furrow openers that work well in heavy or sticky soils. Also, combine harvesters while harvesting paddy in a wet field make the field undulated due to this the sowing of wheat using HS is not at a uniform depth as it is a zero tillage machine. However, in the case of PSS, tillage is done in the seed row, and will this take care of field undulations.

**Table 7:** Wheat yield and yield contributing parameters under different seeding and residue management machines during 2020–2021.

Straw management Seeders	No. of grains/spike	Tiller density (m^−2^)	1000-grain weight (g)	Grain yield (t ha^−1^)
HS	55.2 ± 4.4	383 ± 14.7	41.8 ± 0.5	4.78 ± 0.01
SS	58.8 ± 3.7	369 ± 27.1	39.0 ± 0.9	4.36 ± 0.07
PSS	61.8 ± 1.9	365 ± 12.4	39.4 ± 0.7	4.73 ± 0.33
CT	56.8 ± 4.6	396 ± 28.9	40.8 ± 1.0	4.28 ± 0.10

*PSS* PAU Smart Seeder, *HS* Happy Seeder, *SS* Super Seeder, *CT* conventional till after straw removal.

#### On-farm evaluation of PAU Smart Seeder (PSS) at different locations in 2020–2021

Results from the study showed that the average wheat yield (n = 8) was similar (5.12 v/s 5.10 t ha^−1^) for PSS and HS (Table 8). However, the average wheat yield for the two sites was 4.1% higher for PSS compared to SS (4.71 kg ha^−1^).

**Table 8:** On-farm trials on evaluation of PAU Smart Seeder (PSS), Happy Seeder (HS) and Super Seeder (SS) in wheat sown into rice residues in the rice–wheat system during 2020–21.

Sr. no.	*Seeder type	Sowing date (2020)	Location (district)	Area (ha)	Wheat variety	Wheat yield (t/ha)
1	PSS	04 Nov.	KVK, Samrala (Ludhiana)	0.2	PBW 677	5.04
	HS					5.04
2	PSS	06 Nov.	KVK, Kheri (Sangrur)	0.2	DBW 187	5.13
	HS					4.90
3	PSS	31 Oct.	Surkhpur (Kapurthala)	0.4	HD 3086	5.50
	HS					5.38
4	PSS	31 Oct.	Teerewal (Kapurthala)	0.6	HD 3086	5.63
	HS					5.50
5	PSS	02–03 Nov.	Jaitowal (Kapurthala)	6.0	PBW 725	4.61
	HS					4.55
6	PSS	01–02 Nov.	Mehsampur (Kapurthala)	0.4	Unnat PBW 343	4.45
	HS					4.89
7	PSS	04 Nov.	Thablan (Fatehgarh Sahib)	0.2	PBW 677	5.25
	HS					5.21
8	PSS	09–10 Nov.	Rajoan (Ludhiana)	6.6	HD 3086	5.36
	HS					5.31
9	PSS	04 Nov.	Kedi Bhamal (Ludhiana)	0.4	Unnat PBW 343	4.67
	SS					4.47
10	PSS	07 Nov.	Kattu (Barnala)	0.4	Unnat PBW 343	5.13
	SS					4.94
Average wheat yield PSS versus HS (n = 8)						5.12 vs 5.10
Average wheat yield PSS versus SS (n = 2)						4.90 vs 4.71 (4.1%)

## Discussions

The straw present on the field often builds up in front of the tines of the drill and eventually blocks the tine and frame, causing long delays, uneven seeding rate and depth resulting in patchy stand of plants^25^. The furrow openers of traditional strip till drills are a combination of rotary blade and tine furrow opener. Tines of the strip till drill get clogged when operated in paddy residues, leads to low field capacity and uneven germination of wheat.

**Figure 3:**
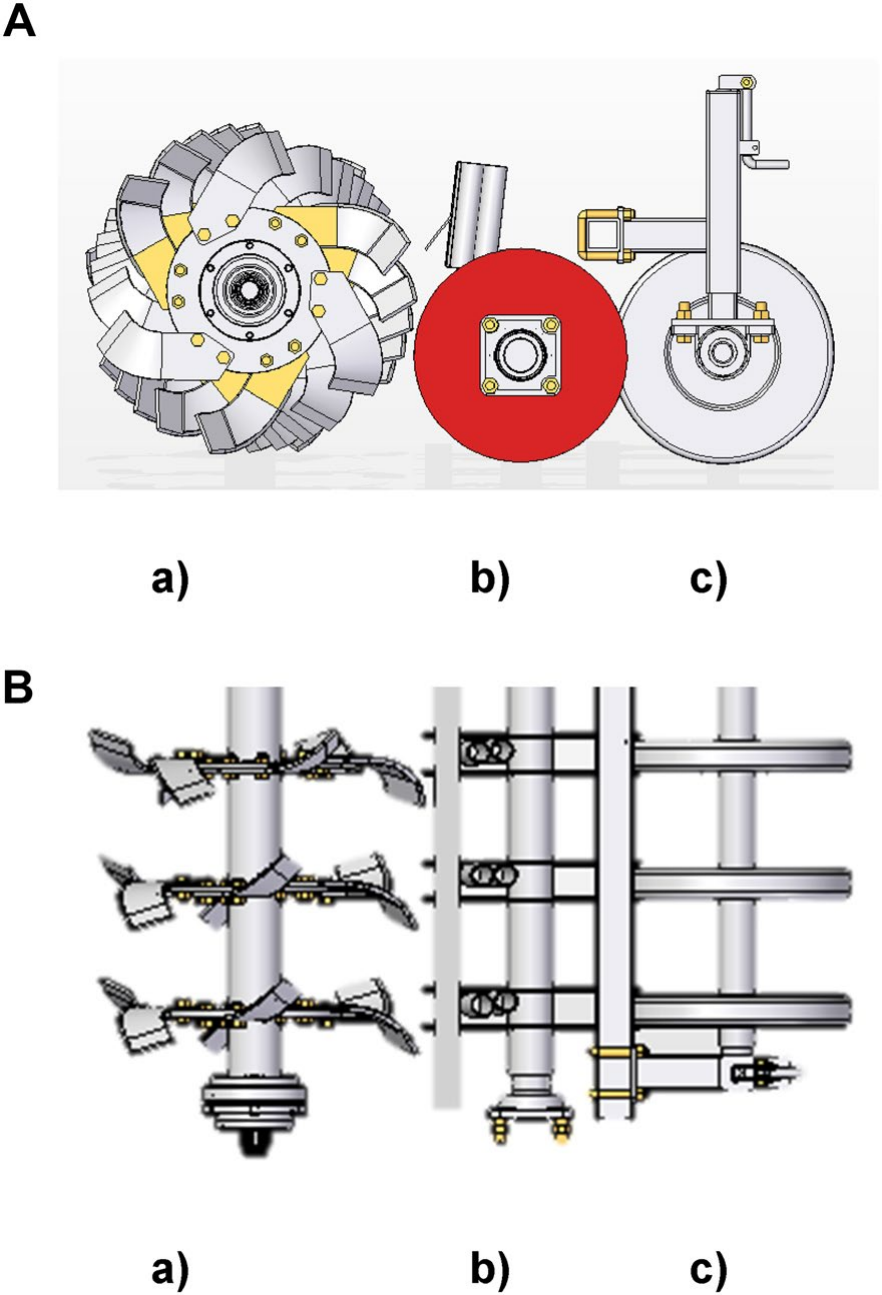
(**A**) PSS furrow opener (a) rotary tillage, (b) passive paired disc and (c) furrow closing roller (side view). (**B**) PSS furrow opener (a) rotary tillage, (b) passive paired disc and (c) furrow closing roller (top view).

The function of furrow openers are (1) to open the furrow, (2) to place the seed and fertilizer in the furrow and (3) to cover the seed and fertilizer with soil to enhance soil-seed contact. In general, the seed placement device, while penetrating into the soil comes in contact with paddy straw and the paddy residues tend to build up on the seed placement device (either furrow opener or tine). PSS furrow opener is unique and different from traditional furrow openers as the function of furrow opener was divided into three different zones one behind another; rotary blades (zone 1), passive paired open discs (zone 2) and furrow closing roller (zone 3). The rotary blades first till a narrow strip of soil and throw the soil into a pair of passive discs, Fig. 3a and b. The seed is dropped inside the passive discs which are mounted on a shaft and enclose the falling seed and fertilizer in a 50 mm wide band. Seed and fertilizer are dropped in the discs from the top, and the rotary action of blades throws the soil inside paired discs. This soil covers the seed and fertilizer dropped in discs. The furrow closing roller follows the passive discs and closes the tilled band. Better soil seed contact leads to early and uniform wheat establishment. Therefore, the absence of tine furrow openers, and mechanical device to place seed and fertilizer into the tilled soil, enables the PSS to get rid of the accumulation of straw residues. The PSS maintains the depth of sowing between 10 to 25 mm in tilled soil strips, which helps in the early emergence of wheat in the residue conditions. The furrow closing rollers move behind the pair of discs and close the tilled soil strip to increase the soil seed contact for the early and uniform establishment of wheat. The furrow closing rollers also act as the depth control wheels for the PSS.

The farmers’ response to PSS was encouraging as the sowing of wheat in paddy residues was smooth. The majority of farmers were satisfied with the performance of PSS over HS as there was no accumulation of paddy straw in-furrow openers of the machine even in wet/heavy soils and better visibility of seed row. This is due to the unique furrow opener design of PSS.

The rotary blade speed of PSS was 127.5 rpm for achieving adequate furrow backfill and soil tilth. The findings^26^ emphasized backfill and furrow tilth to adequately cover seeds to ensure seed emergence and reduce the risk of bird damage in case of strip-till sowing. They reported that a rotary speed of 127 rpm is better for conventional strip till blades. At lesser rotor speed, clod formation reduces the soil tilth whereas, at higher rotor speed the furrow backfill was lesser.

HS is a zero tillage machine, SS is a complete tillage machine, and PSS tills around 32.5% (75 mm wide) of machine width. PSS combines both processes of incorporation and surface retention of residues in one machine; therefore, the fuel consumption of PSS was in between HS and SS. The PSS manages the paddy stubbles partly by surface mulch (in between seed rows), tillage, and incorporation in seed rows. The tillage in seed rows increases the soil seed contact and seed row visibility (after sowing operation) which provides the satisfaction of sowing to the farmers. Moreover, residue retention in between seed rows provides benefits of weed suppression, moisture conservation, and slow decomposition of paddy residues. Thus, PSS is better as compared to HS.

The PSS incorporates only a small part of the straw in the soil and retains the majority of straw as surface mulch due to this the chances of seeds dropping on straw are lesser. On the other hand, the SS does provide a clean look of the field due to which the farmers are attracted to the machine but it pushes the residue into the soil without size reduction (Chopping) and covers the residue with a thin layer of soil. Thus, wheat germination is patchy and lower due to lesser soil seed contact. The germination in the case of PSS is better than SS as the seeds are placed in a well-tilled soil strip having a lesser amount of paddy residues as most of the residues are retained as surface mulch. A study^27^ compared strip tillage with zero tillage using the Combo + Happy Seeder (strip-tillage) and Combo Happy Seeder (zero-tillage), on a sandy loam. They reported that plant density and yield were significantly lower with the zero-till Combo Happy Seeder than with the strip-till Combo + Happy Seeder. The current HS (Turbo version) is a zero tillage drill with a straw management rotor. This was the reason for better plant establishment with PSS over HS. The comparative crop establishment video of wheat in paddy residues after 70 days of sowing using HS, SS, and PSS into 8.1 t ha^−1^ paddy straw (*PR-121*) is available online at https://youtu.be/kd-Zu5jvbRg.

## Conclusions

The study revealed that PSS can operate smoothly under high straw load without any problem of choking of seeding mechanism. Wheat sown using the PSS emerges earlier than when using the existing machines for seeding wheat into combined harvested rice fields. The number of seedlings at 20 days after seeding in PSS treatment was 15.5 and 25.6% higher compared to HS and SS, respectively. The grain yield of wheat was generally higher or similar using PSS over Happy Seeder, whereas, the grain yield of wheat was generally higher for PSS over SS. PSS is more energy-efficient and economical in practice than SS.

PSS has novel mechanism to manage paddy straw and simultaneously sow wheat into heavy straw load (> 8 t ha^−1^) mixture of anchored and loose straw even when the paddy residues become wet during morning and late evening due to dew in winters.

## PSS advantages over HS

1. PSS can work from morning to late evening compared to HS, as it can handle moist paddy straw. The rotary action of PSS strip tillage blades pushes the tractor forward and due to this, it can work in wet fields, which is not possible in the case of tine drills or HS. Therefore, the PSS can work for longer duration in a day compared to HS. This is an important consideration for custom hiring operators as PSS can sow around 10 acres/day whereas HS can sow only 6.0 acres/day.
2. PSS works in conditions where it is difficult to work with HS such as; HS is sensitive to soil moisture of the field. In wet fields, soil in between consecutive furrow openers is sliced and struck in tines which cause the choking of straw in the machine. Moreover, the tractor wheel slippage is excessive which makes sowing ineffective.
3. The wheat establishment in paddy residues is generally less visible in the initial growth stage due to the presence of surface residues in the field. This lesser visibility of wheat establishment is a big bottleneck among farmers’ mindset for the adoption of HS. PSS sows wheat in a band width of 50 mm which is wider than the wheat sown by any other machine. This feature is liked by farmers as it makes germination look broader and appears similar to wheat establishment in residue-free fields.
4. The combine harvester working in wet paddy fields causes ruts due to combined tyre movement. These ruts make the field uneven and zero till machines like HS cannot operate in undulated fields. However, the PSS has rotary tillage blades which clears the undulations of combine tyers and can sow the wheat effectively.

## PSS advantages over SS

1. The average fuel consumption of PSS was 23.3% lesser than SS, whereas, the field capacity of PSS 52% higher than SS. SS consumes more fuel over PSS as it does complete tillage/incorporation of soil/residues for full machine width while PSS tills 37.5% of total machine width.
2. The SS requires high horse power (> 55 hp, double clutch) with a creeper gear having a travel speed of less than 1 kmh^−1^. On the other hand, PSS requires a 50 hp double clutch tractor, generally available with the farmers.
3. The SS can sow only 5 acres/day due to its slower forward speed of travel (< 1 km h^−1^) and 50% lesser field capacity than PSS. It is a main bottleneck for adoption among custom operators of SS.
4. Field looks after SS operation clean/residue free than HS and pleasing site to the farmers. Residue-free look of the field was the main reason for SS exponential adoption over the HS in last two years (2019–2020 and 2020–2021). However, the wheat establishment is delayed and patchy due to the complete incorporation of residues and lesser soil seed contact. On the other hand, PSS provides uniform and early wheat establishment having a clean look of seed row. The tillage in seed rows increases the soil seed contact and seed row visibility (after sowing operation) which provides the satisfaction of sowing to the farmers.
5. In general, tillage increases the weed germination. The weed count and weed biomass for SS were significantly higher by 115.5 and 115% over PSS, respectively.

## Methods

### Description and functions of PAU Smart Seeder

**Figure 4:**
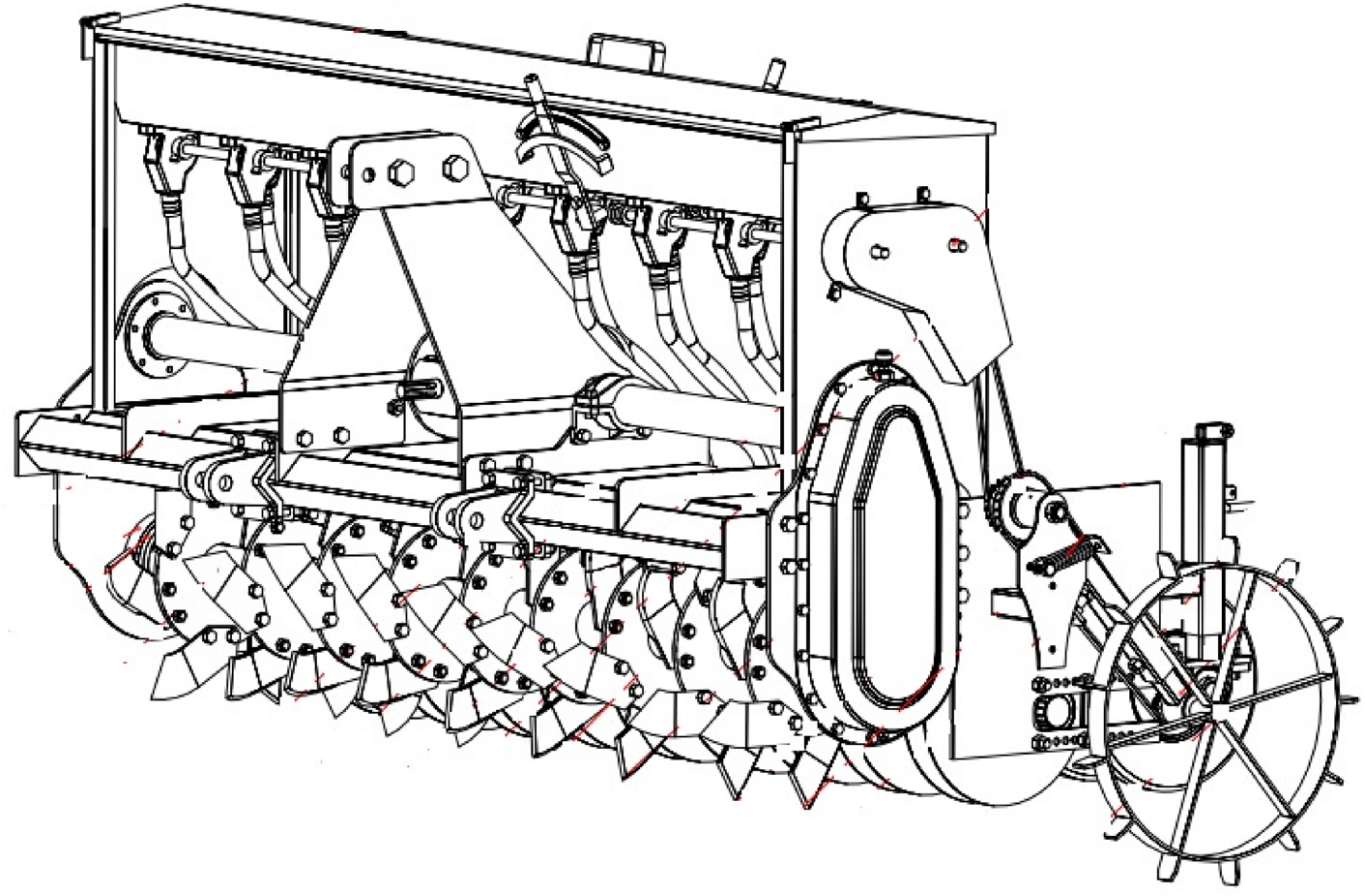
Isometric view of PAU Smart Seeder (front).

**Figure 5:**
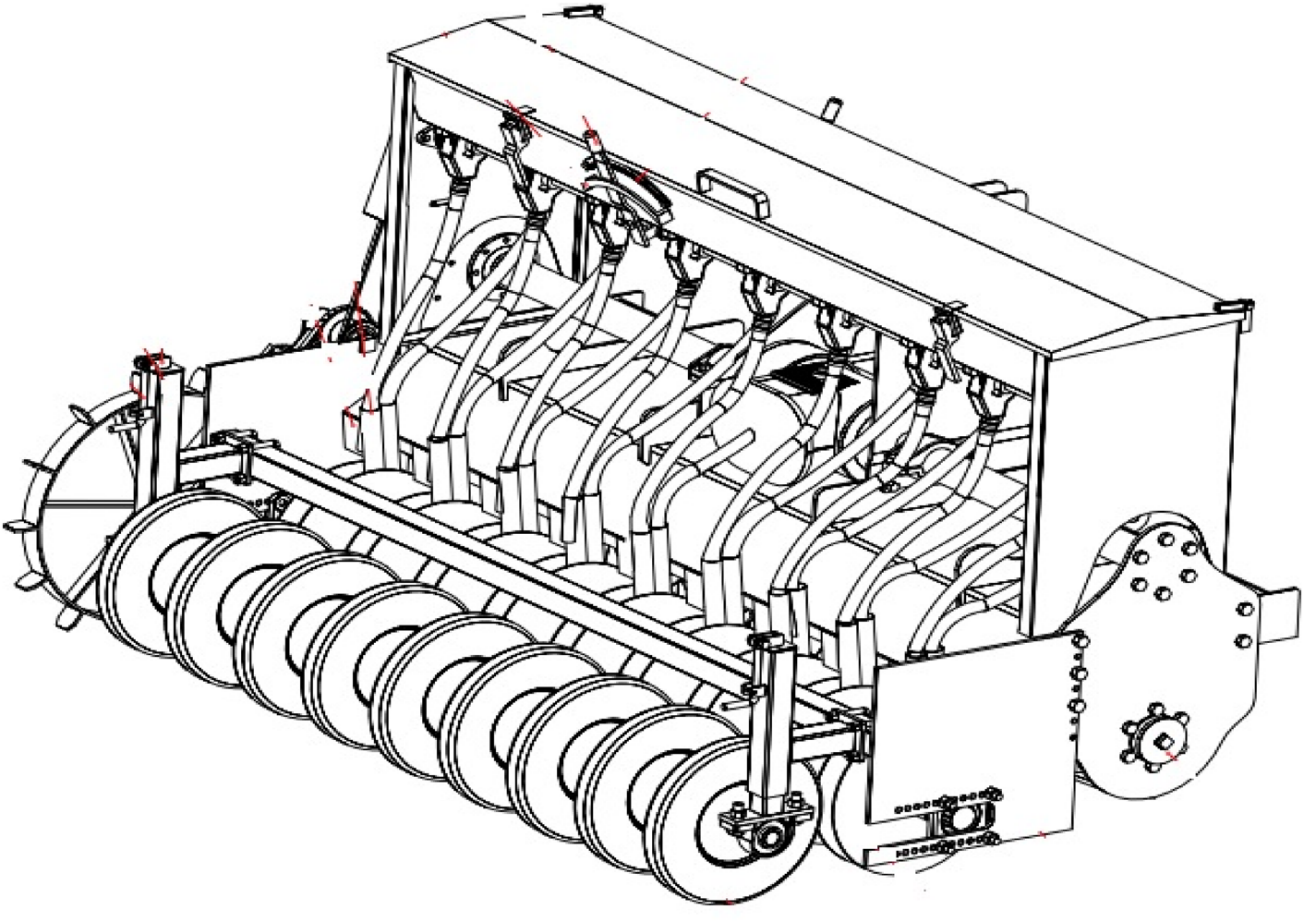
Isometric view of PAU Smart Seeder (rear).

The prototype of PSS was constructed in the workshop of the Department of Farm Machinery and Power Engineering, Punjab Agricultural University, Ludhiana (India). The PSS is a 9-row seed-cum-fertilizer drill consisting of a strip-tillage rotor for managing the rice straw residues and a disc-type seeding mechanism for sowing wheat. With the help of a three-point linkage, the machine hitched to the tractor. The PTO shaft of a tractor is attached to the machine gearbox and gives drive to the strip-tillage rotor through spur and bevel gear arrangement. The strip-tillage rotor consists of nine flanges welded at an equal spacing to a high-pressure MS pipe called a rotor shaft. The flange diameter is 280 mm, and the spacing between two adjacent flanges is the same as the row spacing of the crop to be sown. A set of six curved blades, made from high carbon steel, are bolted to either side of each flange in an alternating pattern such that when they rotate, the blades till a narrow strip of 37.5 mm width on one side of the flange and the adjacent blade till another strip of equal width over the other side of the flange (Figs. 4–5). This rotary action of the blades produces a 75 mm tilled strip ahead of the furrow openers. The loose/anchored straw in front of the blades is chopped and incorporated into the soil, and the straw between two adjacent furrows is left undisturbed as mulch.

Furrow openers on the PSS are pairs of discs fixed on a shaft of 75 mm diameter and attached behind the housing of the strip-tillage rotor assembly (Fig. 4). The spacing between two adjacent furrow openers is the same as the row spacing of the crop to be sown and is generally around 200–220 mm^28^.

The advantage of the disc-type furrow opener is that it works satisfactorily in all kinds of unfavourable conditions as listed for HS under the Introduction section. Where the ground is hard, a fair penetration can be ensured; when there is straw, the disc will cut through it and not carry it; where the tilth is poor, the strip-till rotor and disc will improve seed placement by its pulverizing action; and where it is sticky, the disc will remain clean better than other types of furrow openers^29^.

The best attribute of fixed disc furrow openers is to place all the seeds at the same depth. Seeds and fertilizer are released through the fluted roller mechanisms and move along the seed delivery tube where they are dropped between the double-disc furrow openers and sown at a uniform depth. The seed and fertilizer is placed in a band of 50 cm width in between two fixed disc openers, therefore, in PSS there is no clogging of seed and fertilizer in-furrow openers. Whereas, in the case of HS and SS there are chances of frequent clogging in-furrow openers and it is hard for the farmers to detect the clogging of seed and fertilizer due to the presence of residues. An attachment of a set of 9 cylindrical furrow closing rollers on a shaft of 60 mm diameter is provided behind the disc furrow openers. The width of the rollers is the same as the width of the furrows to be closed, i.e., 50 mm (Fig. 5). The rollers press the seeds and fertilizer, enhancing the seed-soil contact, thus ensuring better crop emergence. They also facilitate balancing the machine, which results in better depth control and smooth operation of the machine. Specification of the PSS is given in Table 9.

**Table 9:** Specifications of the PAU Smart Seeder, Happy Seeder and Super Seeder.

Description or component	Happy Seeder (HS)	Super Seeder (SS)	PAU Smart Seeder (PSS)
Mounting type	Tractor mounted	Tractor mounted	Tractor mounted
Power required (hp)	50	60	50
Transmission system	Tractor PTO (540 rpm)	Tractor PTO (540 rpm)	Tractor PTO (540 rpm)
Weight (kg)	730	1080	810
Sowing unit			
Number of furrow openers	10	11	9
Type of furrow openers	Inverted t-type	Combination of moving disc and tine	Fixed disc type
Row spacing (mm)	225	225	220
Seed and fertilizer metering devices	Fluted feed rollers	Fluted feed rollers	Fluted feed rollers
Straw management unit			
Rotor blade type	Inverted gamma Flails	LJF rotavator blades	J shape blades
Method of mounting on rotor	Hinged	Bolted, fixed	Bolted, fixed
Diameter of rotor with blade	750 mm	458 mm	550 mm
rpm of rotor shaft at 540 rpm of PTO shaft	1000	270	188
Peripheral speed of rotor blades, m/s	39.3	6.5	5.4
Power transmission system for rotor unit			
Primary reduction	Spur and bevel gears combination	Spur & bevel gears combination	Spur & bevel gears combination
Secondary reduction	V-belts	Spur gears	Spur gears

### Experimental design and treatments

#### Experiment 1: Effect of straw load and speed index on the performance of the Smart Seeder

Field experiments were conducted at the Research Farm of the Department of Farm Machinery and Power Engineering, Punjab Agricultural University, Ludhiana, Punjab. The field is located at 30°54ʹ N latitude, 75°48ʹ E longitude, and at an altitude of 245 m above the mean sea level. Performance of the PAU Smart Seeder (PSS) for direct sown wheat in combined harvested rice fields was evaluated and compared with already existing direct seeding machines, i.e., Happy Seeder (HS) and Super Seeder (SS). Rice (variety PR-121) was raised during the Kharif season (June–October) before the sowing of an experimental crop of wheat. The rice crop was harvested using a combine harvester fitted with a straw management system (Super-SMS), and the standing stubbles of approximately 30–40 cm length left over the soil surface, along with loose straw residues.

Ideal furrow quality parameters (such as cross-sectional area, furrow backfill, moisture content, bulk density, and penetration resistance) can be achieved through the appropriate structural design (including type, thickness, cutting edge angle and thickness, rake angle) of the furrow opener^30,31^. These parameters can also be changed with the forward speed of the tractor during planting and the presence of crop stubble. A greater forward speed of the tractor increases the displacement of soil, and crop stubble may lead to poor soil backfill and influence the other parameters^32^. Therefore, the Smart Seeder was operated under different straw loads (4.2, 5.2, and 6.0 t ha^−1^) and at different forward speeds (2.1, 2.6, 3.2 km h^−1^). Rotor rpm at different engine speeds were noted and speed index was expressed as the ratio of the forward speed of operation and rotor rpm. The effects of different straw loads (S) and speed index (R) on various dependent parameters of the machine were determined through field experiments. The experimental field (5000 m2) was divided into three subplots with different straw loads S1 (4.2 t ha^−1^), S2 (5.2 t ha^−1^), and S3 (6 t ha^−1^) adjusted manually. In each section, three sub treatments of forward speeds of PSS were laid down in a randomized complete block design (RCBD) with three replications for each treatment. Wheat (variety PBW 725) was sown during the first week of November 2019. A seed rate of 120 kg ha^−1^ and 60 kg P_2_O_5_ ha^−1^ as diammonium phosphate fertilizer (DAP) were applied at sowing as per the recommendation of the Punjab Agricultural University (PAU), Ludhiana^33^. Fertilizer nitrogen at 120 kg N ha^−1^ (minus the N supplied by DAP) as urea was top-dressed in two equal split doses immediately before the first irrigation (25 to 30 days after seeding) and second irrigation (50 to 55 days after seeding). All other practices for growing wheat crops were followed as recommended by PAU, Ludhiana^33^.

#### Experiment 2: Evaluation of the Smart Seeder vis-à-vis existing rice residue management seeders in 2019–20

Field experiments were conducted at two locations to compare the performance of PSS with the two existing direct seeding machines (HS and SS) which are commonly used by farmers in NW India. The specifications of the three different machines used for the study are provided in Table 1. The performance of the machines was determined in terms of fuel consumption, effective field capacity, seed germination, weed emergence, and grain yield.

Location 1: First field experiment was conducted at Research Farm of the Department of Farm Machinery and Power Engineering, PAU, Ludhiana, India. In the previous season, well-managed rice (variety PR 121) crop was grown in the experiment field. The rice crop was harvested using a combine and the average straw load (dry weight basis) was about 6.0 t ha^−1^. An experimental crop of wheat (variety PBW 725) was sown on 09 November 2019 using the three machines. There were three replications of each treatment (machine) with a plot size of 50 m^2^.

Location 2: Second field experiment was conducted at the PAU Seed Farm, Ladhowal, Punjab located about an 18 km distance from location 1. Rice genotype RYT 3468 was grown in the experiment field and was harvested using a combine. The average rice straw load was recorded as 5.9 t ha^−1^. Wheat variety PBW 1-Zn was sown using three seeders on 18 November 2019. There were three replications and the size of each subplot was 200 m^2^. All the recommended agronomic practices listed under experiment 1 were followed in wheat (Anonymous, 2019).

#### Experiment 3. Comparative performance of different seeders for sowing wheat into rice residue in the rice–wheat system during 2020–2021

A field experiment was conducted for evaluation of three different seeders (PSS, HS, and SS) for sowing wheat into residue after combine harvesting of rice along with additional treatment consisting of conventional till sowing (after rice straw removal). The soil of the experimental field was loamy sand in texture. The rice straw load was 7.1 t ha^−1^. Wheat variety PBW 725 was sown on 11 November 2020 and harvested in the second week of April 2021. The treatments were completely randomized within a block and replicated thrice. Data on grain yield and yield contributing characters were recorded at maturity.

#### Experiment 4. On-farm evaluation of Smart Seeder at different locations in 2020–2021

Performance of wheat sown into rice residue using PSS and Happy Seeder or Super Seeder was compared on farmers’ fields at 10 locations in Punjab state of India. Wheat was sown between 31 October and 10 November 2020. The experiments were conducted in large plots ranging from 0.2 to 0.6 ha each. The experimental details of trials conducted such as; date of sowing, wheat variety, and location are included in Table 8.

### Observations and calculations

#### Straw load (S)

Straw load refers to the weight of rice straw (both standing and loose) present in the experimental field after the combine harvesting of rice crop Three subsamples were collected from randomly selected locations in the main field by placing a 1 m^2^ iron cage. All the loose and standing straw inside the ring were manually collected and weighed. The known weight of subsamples was oven-dried at 60 °C for 24 h. The dry weight of the straw was then recorded for calculating straw load as t ha^−1^.

#### Speed Index (R)

The tractor (50 hp, John Deere 5310) was run at different engine speeds varying from 1500 to 2400 rpm while the PTO was engaged. A digital tachometer (*Mitutoy*) was used to measure the rpm of the machine rotor at these different engine speeds and the observations were noted down. The combinations of engine and rotor speeds in which the machine operated smoothly without choking during the preliminary tests were selected for the subsequent field trials. Forward speeds of operation corresponding to engine rpm and different forward gears (A1 and A2) are shown (Table 10).

**Table 10:** Speed index associated with different combinations of engine rpm, rotor rpm and forward (Fwd) gears.

Engine rpm	Rotor speed (km h^−1^)	Fwd gear A1 Speed (km h^−1^)	Fwd gear A2 Speed (km h^−1^)	Speed index
2000	16.27	2.12	3.27	0.13
1600	13.21	1.73	2.67	0.20
2000	14.20	2.12	3.27	0.23

The speed index was expressed as the ratio of tractor forward speed and rotor speed. Three-speed indexes selected for trials were: R1 (0.13), ratio of forward speed 2.12 km h^−1^ and rotor speed 16.27 km h^−1^; R2 (0.20), ratio of forward speed 2.67 km h^−1^ and rotor speed 13.21 km h^−1^; R3 (0.23), ratio of forward speed 3.27 km h^−1^and rotor speed 14.20 km h^−1^.

1 $$\text{Speed index}=\frac{\text{Forward speed of operation }}{\text{Rotor speed}}$$

#### Fuel consumption

A fuel flow meter (*Aqua metro*, 1 ml) was installed between the diesel tank and fuel filters of the tractor to measure the volume of fuel consumed in the 20 m length of travel. Fuel flow meter reading was noted at the start and end of each test run and the difference between the two readings was taken as the volume of fuel consumed per test run. The lowest count of the fuel flow meter was 1 ml. Fuel consumption was expressed in l h^−1^ (fuel consumption (FC_h_) on hourly basis and it shows load on tractor) and l ha^−1^ (fuel consumption (FC_a_) on per unit area basis and it shows load per unit area basis).

#### Effective field capacity

Effective field capacity (EFC) is the actual rate (ha h^−1^) of coverage by the machine, based on the total field time. It is a function of the rated width of the machine, speed of travel, and the amount of field time lost during the operation (i.e., during headland turning, refilling of seed/fertilizer box, minor adjustments, etc.). EFC was calculated using the formulae given by Kepner et al.^34^ (Eq. 2).

2 $${\text{EFC}} = \frac{{{\text{w}} \times {\text{s}}}}{{10}} \times \eta$$

where, W = effective width of machine (m), S = forward speed of the machine, in km h^−1^, $\eta$ = field efficiency, assumed as 70%.

#### Wheel slip

Tractor drive wheels slip in all field operations such that the distance covered by the tractor in a given number of drive wheel revolutions decreases with the wheel slip. Distance travelled by tractor in the field with and without load (machine), in four revolutions of the drive wheel, was measured. The Wheel slip was calculated as follows (Eq. 3):

3 $$\% \text{wheel slip }= \frac{\text{D}1-\text{D}2}{\text{D}1} \times 100$$

where, D1 = distance covered in 4 revolutions without load, D2 = distance covered in 4 revolutions with load.

#### Seedling emergence

Seed germination (seedlings that had emerged through the soil) was noted 20 days after sowing. The number of seedlings per meter row length in 5 adjacent rows was recorded in each plot and their average value was calculated, and then converted to the number of seedlings m^−2^ for all treatments.

#### Weed density

Weed emergence in terms of weed density (number of weeds/m^2^) was recorded from two randomly selected locations within each plot using 1 m^2^ quadrate at 30 days after sowing. All the weed species (grasses, broad leaves, and sedges) that fell inside the quadrates were counted. The weed density was calculated as under;

4 $$\text{Weed density }=\frac{\text{Total count of weeds inside quadrate}}{\text{Area of quadrate }{(\text{m}}^{2})}$$

#### Weed biomass

The weed samples taken for weed count were used for weed dry matter. The weeds were washed in water and kept under sunlight for few hours for partial drying, then dried in a hot air oven at 72 ºC for 48 h to a constant weight^35^. Samples were kept in desiccators for cooling and their dry weight was recorded using an electronic balance. Weed biomass was reported as g m^−2^.

#### Grain yield

An area measuring about 2 to 2.25 m^2^ was marked (5 wheat rows and 2 m length for all different machines) with the help of a measuring tape in each plot and all plants falling in the marked area were manually harvested. The harvested wheat was threshed using a small plot power thresher. The grain moisture content of wheat was determined by oven drying subsamples at 60 °C to a constant weight, and grain yield was expressed at 12% grain moisture content.

#### Yield attributes of wheat

Wheat yield contributing characters such as spike density (number of effective tillers per meter square), grains per spike, and 1000-grain weight were recorded at maturity. The number of spikes per meter row length was measured from two adjacent rows within each plot to determine spike density. Spike length was measured from randomly selected 10 plants within each plot. Thousand-grain weight was determined from a subsample collected from the bulk sample of each plot.

#### Energy consumption for the establishment of wheat

The data on crop management inputs such as the number of tillage, fuel consumption, number of irrigations, herbicide, fertilizer, seed rate, labour use, pesticide application, and their costs under each treatment were recorded for each crop using a standard data recording format. The energy consumption for sowing operation of wheat using the PSS, HS, SS, and farmer’s practice (FP) was calculated from the sum of direct (e.g., human labor, diesel, electricity) and indirect (energy embodied in machinery) energy consumption^9^. The PSS, HS, and SS sow wheat in single machine operation, and FP consisted of the use of stubble shaver for residue slashing, disc harrow (1), cultivator (1), and planker (2) for tillage and seedbed preparation followed by one pass of seed –cum-fertilizer drill for sowing of wheat.

The values of the energy equivalent for each form of energy consumption are provided in Table 11, and the parameters and assumptions used to calculate energy expenditure for each farm operation are included in Table 12. Energy consumption in machinery operations was computed using the actual rate (ha h^−1^) of coverage by the machine and the total field time consumed. It is a function of the rated width of the machine, speed of travel, and the amount of field time lost during the operation (i.e. during headland turning, refilling of seed/fertilizer box, minor adjustments, etc.).

**Table 11:** Values of energy equivalents for different sources of energy^36^.

Source	Energy equivalents
Human	1.96 MJ h^−1^
Diesel	56.31 MJ l^−1^
Electricity	11.93 MJ kw h^−1^
Tractor	64.8 MJ h^−1^
Machinery	62.7 MJ h^−1^

**Table 12:** Assumptions and parameters considered for calculating energy expenditure for different farm operations.

Source	Width of machine (m)	Machine weight (kg)	Wear out life (h)	Human labour (h ha^−1^)	Effective field capacity^#^ (ha h^−1^)	Fuel consumption^#^ (l h^−1^)
Tractor (37.3 kw)	–^a^	2000	10,000	–^a^	–^a^	–^a^
HS	2.25	680	2400	5.26	0.38	4.5
PSS	2.20	700	2400	5.26	0.38	5.72
SS	2.25	980	2400	8	0.25	6.77
Seed-fertilizer drill	2.6	275	2400	2.56	0.78	3.5
Stubble shaver	1.78	200	4000	1.25	0.8	3.5
Disc harrow	2.6	350	3000	1	1	5
Cultivator	2.6	200	4000	1	1	5
Planker	3	150	4000	0.71	1.4	3.5
Residue burning	–	–	–	1	–	–
Rodent control	–	–	–	1	–	–
Irrigation pump (11.25 kw)	–	100	10,000	10	0.1	–
Straw spreading (Super SMS)	4.27	175	4000	1	1	2.5

–^a^These values of tractor were included in the respective farm machinery operations.

^#^Data in consultation with Farm Machinery test centre, PAU, Ludhiana (India).

The human, fuel, and machinery energy consumption (MJ ha^−1^) for each operation was determined using Eqs. (5) to (7).

5 $$\text{Human energy}, {\text{MJ ha}}^{-1}= \frac{\text{Number of labour used }\times \text{time},\text{ h}}{\text{Area covered},\text{ ha}} \times \text{energy equivalent}, {\text{MJ h}}^{-1}$$

6 $$\text{Fuel energy}, {\text{MJ ha}}^{-1}= \frac{\text{Fuel Consumption}, {\text{ l h}}^{-1}}{\text{Effective field capacity}, {\text{ ha h}}^{-1}} \times \text{energy equivalent}, {\text{MJ l}}^{-1}$$

7 $$\text{Machine energy}, {\text{MJ ha}}^{-1}= \frac{\text{weight of machine},\text{ kg}}{\text{Wear out life},\text{ h }\times \text{ effective field capacity}, {\text{ ha h}}^{-1}} \times \text{energy equivalent}, {\text{MJ h}}^{-1}$$

Similarly, electric energy for pumping water was calculated from the amount of electricity consumed, kWh, the rate of irrigation, ha h^−1^, and the energy equivalent of electricity.

### Statistical analysis

Statistical analysis of data (using SAS v 9.4) was performed using a randomized block design to determine the effect of straw load and speed index on dependent variables^37^. Comparison of the means was made using the least significant difference (LSD) test and statistical significance was declared for means differing at P < 0.05. A randomized complete block design was used to analyze the difference between two treatment means (machines) on different crop and weed parameters in experiment 2. The difference between the two treatment means was compared using Tukey Test at p < 0.05.

### Supplementary Information

Supplementary Information.

## Data Availability

The datasets used and/or analyzed during the current study are available from the corresponding author on reasonable request.
