# Supplementary material for: PAU Smart Seeder: a novel way forward for rice residue management in North-west India
Source: Sci Rep. 2024 May 23;14:11747. doi: 10.1038/s41598-024-62337-z (PMC11111448; doi:10.1038/s41598-024-62337-z)
Supplement: Supplementary file 1 — Supplementary Information. [file 41598_2024_62337_MOESM1_ESM.docx]

| 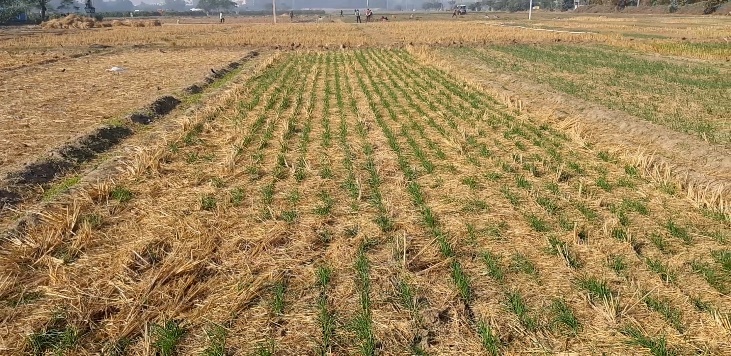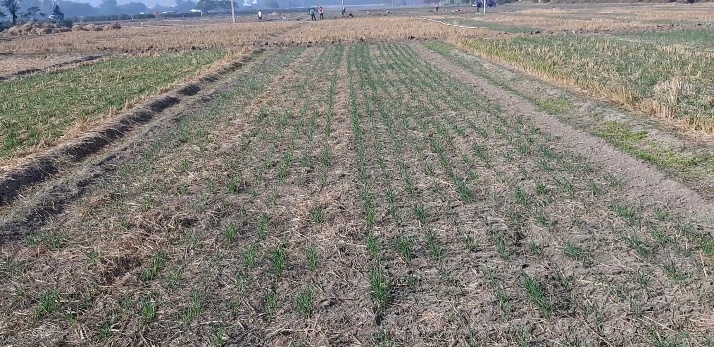   1. **Happy Seeder b) Super Seeder** |
| --- |
| 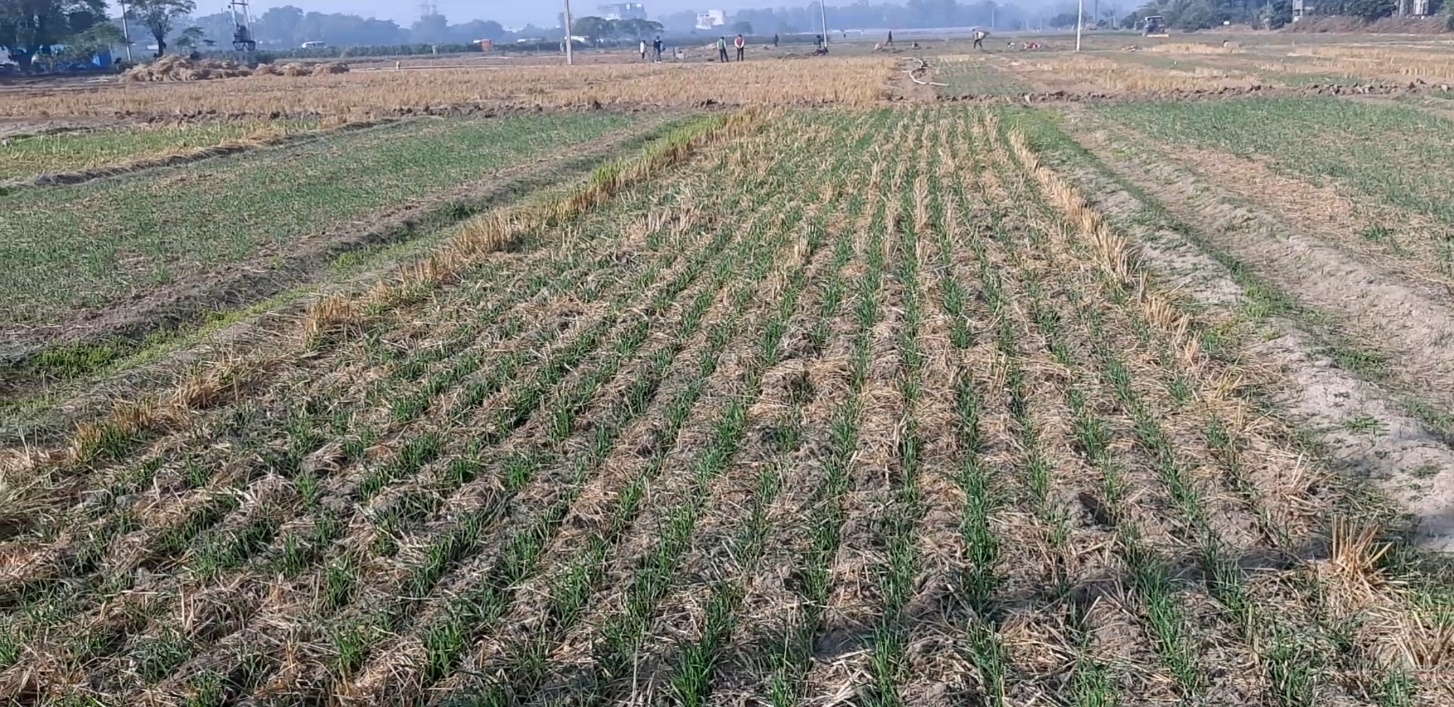   1. **PAU Smart Seeder** |

**Figure 1s. Crop establishment after sowing with Happy Seeder (a), Super Seeder (b) and PAU Smart Seeder (c)**
